# Contralateral Prophylactic Mastectomy among Women with Pathogenic Variants in *BRCA1/2*: Overall Survival, Racial, and Ethnic Differences

**DOI:** 10.1155/2022/1447545

**Published:** 2022-12-31

**Authors:** Sukh Makhnoon, Angelica M. Gutierrez Barrera, Roland Bassett, Aimaz Afrough, Isabelle Bedrosian, Banu K. Arun

**Affiliations:** ^1^Department of Behavioral Science, UT MD Anderson Cancer Center, Houston, TX, USA; ^2^Department of Breast Medical Oncology Research, UT MD Anderson Cancer Center, Houston, TX, USA; ^3^Department of Biostatistics, UT MD Anderson Cancer Center, Houston, TX, USA; ^4^Department of Internal Medicine, Hematology/Oncology, UT Southwestern Medical Center, Dallas, TX, USA; ^5^Department of Breast Surgical Oncology, UT MD Anderson Cancer Center, Houston, TX, USA

## Abstract

**Background:**

Patients with unilateral breast cancer carrying pathogenic variants in *BRCA1/2* have the option to undergo contralateral prophylactic mastectomy (CPM). However, differences in CPM use and survival outcomes following CPM are poorly understood in this high-risk population, in part due to a lack of data from contemporary clinical cohorts. The objective of this study was to evaluate post-CPM overall survival (OS) and related racial/ethnic differences in a contemporary clinical cohort.

**Methods:**

We retrospectively reviewed the medical records of women with a personal history of unilateral breast cancer carrying pathogenic variants in *BRCA1/2* who were diagnosed between 1996 and 2012. Genetic test results, self-reported demographic characteristics, and clinical factors were abstracted from electronic medical records.

**Results:**

Of 144 BRCA-positive patients, the majority were White (79.2%, *n* = 114). Overall, 56.1% (*n* = 81) of all *BRCA1/2* carriers chose to undergo CPM, with no racial/ethnic difference in CPM election (*p* = 0.78). Of 81 patients who underwent CPM, there is strong evidence of a difference in survival between the racial/ethnic groups, with White patients having the highest OS compared to non-White patients (*p* = 0.001). Of the 63 patients who did not undergo CPM, there is no racial/ethnic difference in overall survival (*p* = 0.61). In multivariable cox regression, adjusted for demographic and clinical characteristics, OS was significantly lower among non-Whites than in Whites (HR = 0.39, *p* = 0.04).

**Conclusions:**

Evaluation of a contemporary clinical cohort of BRCA-positive women with unilateral breast cancer showed no racial/ethnic difference in CPM use, but there was a significant difference in post-CPM overall survival.

## 1. Introduction

The risk of contralateral breast cancer among carriers of germline pathogenic variants in *BRCA1/2* with a primary diagnosis of breast cancer [1] is 62–83% higher than in women not carrying these mutations over their lifetimes [2]. This substantial cancer risk can be reduced significantly through contralateral prophylactic mastectomy (CPM). Several professional guidelines including those from the American Society of Breast Surgeons (ASBrS), the American Society of Clinical Oncology (ASCO) [3], and the National Comprehensive Cancer Network (NCCN) support consideration of CPM among *BRCA1/2* positive women due to its demonstrated effectiveness in managing secondary breast cancer risk in terms of life years and quality-adjusted life years gained and cost saving when compared with breast cancer screening. Mirroring these evidence and recommendations, 92% of physicians surveyed from the National Accreditation Program for Breast Centers reported that BRCA deleterious mutation carrier status is the most common indication for them to recommend CPM [4].

The use of CPM has increased dramatically over the past two decades from 3.9% in 2002 to 12.7% in 2012 [5]. The use of germline genetic testing for breast cancer susceptibility has also concomitantly increased over the same time beginning with the discovery of the *BRCA1* gene (in 1994) and the *BRCA2* gene (in 1995). Yet, many large studies include patients from the 1970s and 1980s who were diagnosed and treated for breast cancer before the clinical genetic testing era [6–8] and do not contain information on germline BRCA mutation status [9], which is a strong indication for CPM. Therefore, we cannot assume that the use of CPM and CPM-related outcomes reported from these cohorts is representative of women in current oncology practice who routinely undergo genetic testing. Although racial/ethnic differences in CPM use is well demonstrated in the literature, with higher use of CPM among White women than in non-White women, whether this racial/ethnic difference persists after adjustment for women's BRCA status is less well understood [10]. Up-to-date empirical data that include BRCA genetic test results are needed to investigate sociodemographic differences in CPM uptake and its effect on long-term prognosis.

There is scarce, inconsistent, and generally less compelling evidence on the overall survival advantage resulting from CPM, which was also established by a 2010 Cochrane systematic review of 39 studies [11]. This is likely because most contralateral breast cancers are detected early and prove to be curable. Furthermore, the survival advantage may be a result of selection bias, whereby healthier, younger women are recommended for or chose to undergo CPM resulting in better overall survival [12, 13]. To date, four observational cohort studies have explored post-CPM survival among women carrying germline pathogenic variants in *BRCA1/2*. While one study found no survival advantage after adjustment for oophorectomy [14], three others demonstrated an increase in overall survival [15, 16] as well as breast cancer-specific survival [7] compared to surveillance. However, once again, these three studies included women diagnosed in the 1970s and 1980s, when BRCA genetic testing was unavailable and the routine surveillance modality was mammography, rather than high-risk screening MRI, which is better at early detection for high-risk populations. Thus, it is necessary to establish the survival advantage of CPM (if any) using contemporary cohorts of BRCA-positive women who have the option for screening MRI as an alternative to CPM. In addition, racial/ethnic difference in long-term outcomes of CPM among BRCA-positive women remains underinvestigated partly due to the limited number of large datasets with clinical information that are amenable to such investigation.

This study evaluates overall survival (OS) following CPM among women carrying germline pathogenic variants in *BRCA1/2* with a personal history of unilateral breast cancer in a contemporary cohort. Furthermore, the study aims to understand racial/ethnic differences in CPM use and post-CPM survival. These empirical data on CPM use and survival among the racially and ethnically diverse clinical population can inform the development of clinical practice guidelines as well as cancer prevention and control efforts.

## 2. Materials and Methods

### 2.1. Study Population and Data Collection

This study included women carrying pathogenic variants in *BRCA1/2* with unilateral breast cancer identified through a prospectively maintained clinical database who were seen at the University of Texas MD Anderson Cancer Center between January 1996 and June 2012. Data were retrospectively reviewed to identify eligible women who had undergone clinical genetic testing, carried a pathogenic or likely pathogenic variant in *BRCA1/2*, and had a personal history of unilateral breast cancer. Patients were followed until July 2020. We excluded women who had bilateral breast cancer or metastatic breast cancer.

We reviewed the electronic medical records (EMR) for data including self-reported sociodemographic information: race/ethnicity (non-HispanicWhite, non-HispanicBlack, and Hispanic), marital status (single, married, divorced, and widowed), and education level (high school or less, some college-technical school, university, advanced degree, and higher degrees). Clinical information included breast cancer stage, nodal status, estrogen receptor and progesterone receptor (i.e., hormone receptor status), and Her2/neu status, age at tumor diagnosis, election of CPM; genetic information including the BRCA germline genetic test result, first-degree family history of breast cancer, and first-degree family history of ovarian cancer; and survival status. Women with pathogenic variants in *BRCA1/2* who do not opt for CPM are recommended to undergo screening MRIs as part of usual care at MD Anderson. However, data on screening MRI use was not collected for this study. This study was approved by the University of Texas MD Anderson Cancer Center Institutional Review Board (RCR06-0561).

### 2.2. Statistical Analysis

Patients were characterized with respect to the following demographic and clinical characteristics: age at cancer diagnosis, diagnosis year, marital status (married vs. other), education (less than high school vs. at least high school), first-degree family history of breast cancer (yes vs. no), first-degree family history of ovarian cancer (yes vs. no), stage at diagnosis (I, II, and III; IV was excluded), TNM nodal status (0, 1, 2, 3, and NA), and CPM status (yes vs. no). All patients with a missing clinical stage were excluded from the analyses.

The mean, median, standard deviation, and minimum/maximum values were used to describe continuous variables, and *N* (%) was used to obtain for categorical/ordinal variables. Differences in demographic and clinical features between race cohorts were assessed using Wilcoxon rank sum tests for continuous variables and Fisher's exact tests for categorical variables. The method of Kaplan and Meier was used to estimate the distribution of overall survival (OS) from the date of diagnosis of primary breast cancer to death; patients not known to have died were censored at their last follow-up visit. Cox proportional hazards regression analysis was used to assess the association between OS and demographic and clinical factors of interest. Logistic regression analysis was used to assess the association between CPM and the same factors. In cases where the number of patients in one or more categories was small, Firth's penalized likelihood method was used to fit regression models. All statistical analyses were performed using R version 4.1.1. All statistical tests used a significance level of 5%. No adjustments for multiple testing were made.

## 3. Results

### 3.1. Patient Characteristics

A total of 144 women with a history of unilateral primary breast cancer carrying pathogenic BRCA variants were included in this study. Overall, study participants were predominantly well-educated and married, with a median age of 40 years at breast cancer diagnosis (range: 21–84 years). The majority of the patients were White (79.2%, *n* = 114), and the remaining 10.8% were non-White (Table 1). Race/ethnicity cohorts (White and non-White) were comparable with regard to breast cancer stage, family history of breast and ovarian cancer, HR status, and HER2/neu status. The diagnosis year differed significantly by race/ethnicity (*p* = 0.002), with relatively more white patients diagnosed before 2007 than the other racial groups. The median follow-up was 7.9 years, with a range of 1.62 to 17.4 years.

### 3.2. Selection of CPM

Overall, 56.2% (*n* = 81) of all BRCA-positive women chose to undergo CPM. The rate of CPM election did not differ significantly by race/ethnicity with CPM rates of 56.1% and 56.7% among White and non-White patients, respectively, (*p* = 0.96).  Table 2 summarizes results from univariate logistic regression models for CPM. Of the demographic and clinical variables that were tested, none were significantly associated with CPM uptake.

### 3.3. Survival Analysis

**Figure 1:**
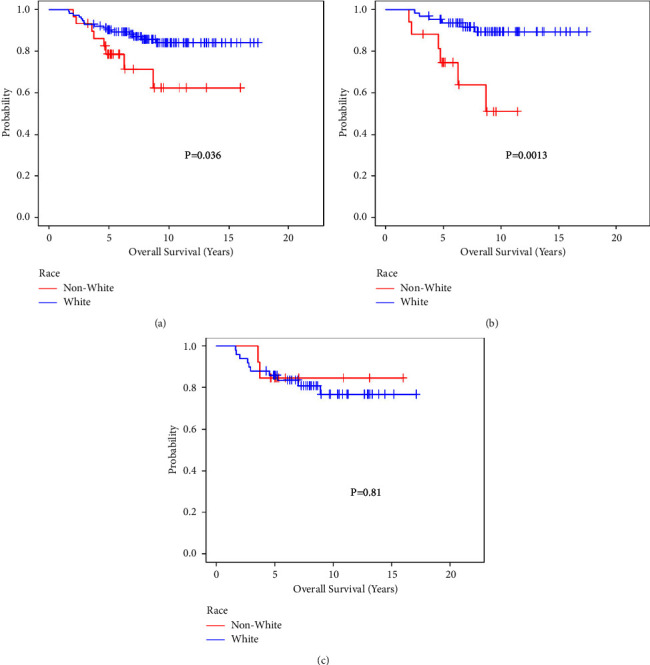
Overall survival by race/ethnicity among (a) all patients (*n* = 144), (b) patients with contralateral prophylactic mastectomy (*N* = 81), and (c) patients without contralateral prophylactic mastectomy (*n* = 63).

**Figure 2:**
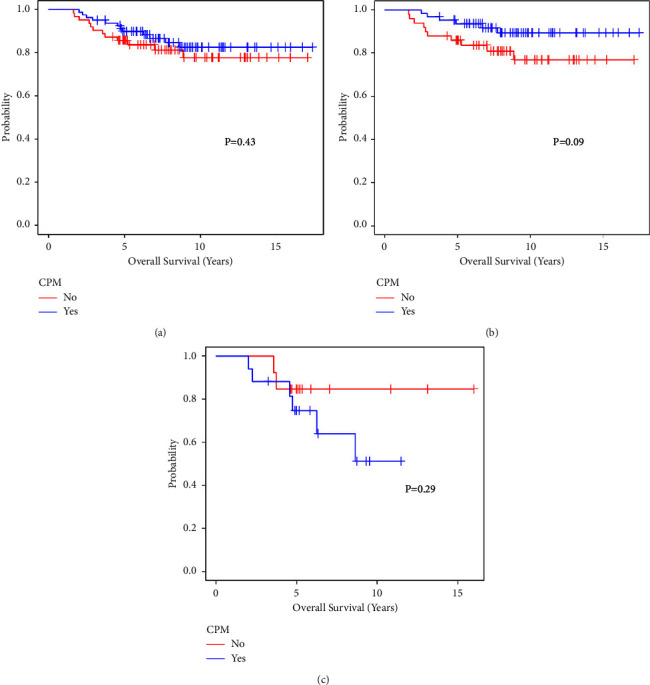
Overall survival comparing (a) patients by contralateral prophylactic mastectomy election and stratifications for (b) white patients and (c) nonwhite patients.

Figure 1 presents the Kaplan–Meier plots of unadjusted overall survival for the BRCA-positive women with and without CPM by race/ethnicity. There is strong evidence of difference in survival between the racial/ethnic groups in the overall sample (*p* = 0.03) driven by 81 patients who underwent CPM: White patients had higher overall survival than non-White patients (*p* = 0.001). Of the 63 patients who did not undergo CPM, there is no difference in overall survival (*p* = 0.81) between the racial/ethnic groups.  Figure 2 shows the overall survival by CPM as well as stratifications by race/ethnicity. There is no evidence of a difference in survival between patients with and without CPM in the overall population (*p* = 0.43) or among Whites (*p* = 0.09) or non-Whites (*p* = 0.29).

Table 3 presents the results of univariable and multivariable Cox regression analyses evaluating predictors of overall survival. In the unadjusted analyses, earlier year of cancer diagnosis (HR = 1.22, *p* = 0.01) and lower clinical stage (compared to stage I, stage II HR = 1.59, and stage III/IV HR = 1.38, *p* = 0.04) were independently associated with overall survival in the unadjusted analyses. After controlling for other covariates, these associations were no longer significant in the multivariate model. However, after adjusting for covariates, non-Whites had a 61% higher hazard of death than Whites (*p* = 0.04).

## 4. Discussion

Our evaluation of a racially/ethnically diverse cohort of women with unilateral breast cancer with pathogenic variants in *BRCA1/2* showed no racial/ethnic difference in CPM use, but there was a higher post-CPM hazard of death among non-Whites than among Whites. In our study, 56.2% of the overall sample elected to undergo CPM, which is comparable to previously reported CPM rates among BRCA carriers that range between 42.3% and 75% [7, 14], [17, 18]. We observed a racial/ethnic difference in the 20-year overall survival among women who underwent CPM after adjusting for relevant covariates including stage, HR status, age at diagnosis, and family history of breast and ovarian cancer.

The lack of racial/ethnic difference in CPM use in our study differs from previous population-based [19] and single-institution studies, including those from this institution [20], which reported significantly higher CPM use among white women than among other racial/ethnic minorities [21–23]. However, in contrast to many of these studies, our study specifically evaluated BRCA-positive women. Variations in access to care including health insurance are hypothesized to be the primary driver of racial/ethnic disparity in CPM use, which is an expensive procedure often followed by costly reconstructive surgery. Thus, social- and system-level factors which underlie disparities in cancer treatment are thought to overshadow any difference in biological determinants for undergoing CPM (ER status and earlier cancer stage). Notably, these cancer-related factors did not differ among patients of different racial/ethnic groups included in our study. Women included in our analysis were seen at a tertiary cancer center where a coordinated team of physicians cares for a largely well-insured patient population. This creates a homogeneous care setting, which likely explains the lack of racial/ethnic disparity in CPM use. This setting is notably different from studies that analyze population-level data, where there is significant heterogeneity in cancer care settings and insurance status. Furthermore, existing population-level studies of disparities in CPM use do not adjust for BRCA mutation status as genetic data are unavailable in most administrative and research datasets in the US.Population-level research is needed to better understand racial/ethnic disparities in CPM use, overall survival, and their predictors.

This study found no evidence of CPM-mediated improvement in overall survival. The expected mechanism of action for CPM-mediated improvement in overall survival is through decreasing the risk of contralateral breast cancer and subsequent cancer mortality. However, contralateral breast cancer is often detected early among BRCA carriers as they undergo frequent surveillance, and the early-stage cancer often proves to be curable. It is therefore unlikely that CPM itself improves survival. However, we know that healthier, well-insured women are more likely to undergo CPM. Any improvement in post-CPM survival is likely a result of the patients' improved access to healthcare throughout their lifetime, as shown by improvement in all-cause mortality in previous studies of this cohort [12], rather than the reduced risk of contralateral breast cancer. In contrast to our results, the four studies that showed improved BRCA-adjusted overall survival after CPM [7, 14, 15, 24] were conducted outside the US, included a patient population that lacked racial/ethnic diversity, and included older patient cohorts than the present study. Therefore, this study is an important addition to the literature on survival advantage of CPM as it is the first US, study of a racially/ethnically diverse cohort that reports a lack of survival advantage. Although the median follow-up time in this study (7.9 years) is comparable to the four previous studies that range between 3.5 and 14.3 years, it is possible that the follow-up is not long enough to detect a significant survival advantage. Women with pathogenic variants in *BRCA1/2* diagnosed with breast cancer have up to 83% risk of developing contralateral breast cancer, which can take an average of 5.7 years to develop. As a result, breast cancer-specific survival advantages will take some time to observe in this cohort. Still, our results align with the results of a meta-analysis that found no evidence of BRCA-unadjusted overall survival after CPM [10] and parallel to those of a modeling study of BRCA carriers that reported a very modest (to negligible) improvement in overall survival [25].

Lack of survival advantage does not suggest that high-risk BRCA-positive women should not undergo CPM, as surgery can have benefits besides survival, including reducing objective risk and worry of ipsilateral breast cancer. A desire for breast symmetry following unilateral breast cancer, associated self-image, and perhaps future sexual encounters may be other reasons that can drive CPM decisions in this cohort. The preferential use of CPM over surveillance among NH White women demonstrated in previous studies [9, 21, 26] is not simply a case of medical gluttony as it comes with significant health risks including complications from surgery and reconstruction and must be weighed against its costs, including those of the procedure, complications, reconstruction, and perhaps psychotherapy. CPM is very much a preference-sensitive decision that requires balancing psychosocial and medical considerations.

Non-White women in this study were found to have an increased hazard of death compared to White women that is not explained by the available clinical or sociodemographic factors. Large studies with detailed data on race and ethnicity as well as breast cancer treatment (e.g., neoadjuvant chemotherapy), adherence, and immediate vs. delayed CPM are needed to better understand and reduce this disparity. Findings from this work should be considered in light of several limitations. Data on complications from CPM or reconstructive surgery and other comorbidities that could impact overall survival were unavailable and not included in our analyses. Patients included in our analyses were well-insured and thus do not represent all women with unilateral breast cancer who may or may not be able to undergo CPM due to lack of insurance coverage. Future research should explore population representative datasets to study racial/ethnic differences in CPM use and associated predictors. A recent study of nearly 460,000 women showed that, compared to NH White women, NH Black and Hispanic women have an increased risk of contralateral breast cancer that is not explained by available clinical or socioeconomic factors that did not include germline mutation data [27]. Future studies should explore racial/ethnic differences in predisposition to contralateral breast cancer and the consequent need for CPM use. In addition, low-penetrance breast cancer susceptibility genes conferring lower lifetime risk of contralateral breast cancer should be explored to understand how they affect the uptake of CPM.

In summary, 56.2% of women carrying *BRCA1/2* mutations in this cohort chose to undergo CPM. Analyses of the clinical cohort of women showed no racial/ethnic difference in CPM use. Reasons underlying this lack of difference may include the largely well-insured women with access to a tertiary cancer hospital who were included in this cohort, but additional risk factors including socioeconomic status and other comorbidities are needed to thoroughly understand drivers of CPM use as they can inform clinical practice guidelines as well as cancer prevention and control efforts. We observed lower post-CPM overall survival among non-Whites than among Whites, which needs to be better understood and addressed using disaggregated race/ethnicity data and additional clinical factors including treatment and adherence that may influence survival.

## Figures and Tables

**Table 1:** Patient characteristics by race/ethnicity (*N* = 144).

Variable	Levels	Whites, *n* = 114 (%)	Nonwhites, *n* = 30 (%)	All *N* = 144 (%)	*p* value	
Age at diagnosis	Median, years	40	39.5	40	0.36	

Diagnosis year	Median					

Marital status	Married	83 (72.8)	20 (66.7)	103 (71.5)	0.5	
	Other	31 (27.2)	10 (33.3)	41 (28.5)		

Education	≥High school	85 (81.7)	17 (63.0)	102 (77.9)	0.07	
	<High school	19 (18.3)	10 (37.0)	29 (22.1)		

Family history of breast cancer	No	60 (53.1)	12 (41.4)	72 (50.7)	0.3	
	Yes	53 (46.9)	17 (58.6)	70 (49.3)		

Family history of ovarian cancer	No	99 (87.6)	24 (82.8)	123 (86.6)	0.54	
	Yes	14 (12.4)	5 (17.2)	19 (13.4)		

Stage	I	35 (30.7)	7 (23.3)	42 (29.2)	0.2	
	II	51 (44.7)	19 (63.3)	70 (48.6)		
	III	28 (24.6)	4 (13.3)	32 (22.2)		

Nodal status	Node 0	57 (50.0)	14 (46.7)	71 (49.3)	0.17	
	Node 1	35 (30.7)	10 (33.3)	45 (31.2)		
	Node 2	7 (6.1)	5 (16.7)	12 (8.3)		
	Node 3	15 (13.2)	1 (3.3)	16 (11.1)		

CPM	No	50 (43.9)	13 (43.3)	63 (43.8)	0.1	
	Yes	64 (56.1)	17 (56.7)	81 (56.2)		

HR status	ER−/PR−	59 (52.2)	12 (40.0)	71 (49.6)	0.3	
	ER+ or PR+	54 (47.8)	18 (60.0)	72 (50.4)		

HER2/neu status	Negative	98 (87.5)	27 (90.0)	125 (88.0)	0.1	
	Positive	14 (12.5)	3 (10.0)	17 (12.0)		

HR: hormone receptor; CPM: contralateral prophylactic mastectomy.

**Table 2:** Univariate logistic regression models for contralateral prophylactic mastectomy.

Parameter	Level	Total *N*	*N* w/CPM	Unadjusted model		
				OR	95% CI	*p* value
Age	Continuous	144	81	1.00	0.96–1.03	0.79

Diagnosis	Year	144	81	0.98	0.89–1.08	0.71

Race	Nonwhites	30	17	Ref		0.96
	Whites	114	64	0.98	0.44–2.20	

Marital status	Married	103	54	Ref		0.14
	Other	41	27	1.75	0.83–3.71	

Education	<High school	102	57	Ref		0.95
	≥ High school	29	16	0.97	0.42–2.23	

Family history of breast cancer	No	72	37	Ref		0.23
	Yes	70	43	1.51	0.77–2.94	

Family history of ovarian cancer	No	123	71	Ref		0.40
	Yes	19	9	0.66	0.25–1.74	

Stage	I	42	24	Ref		0.25
	II	70	43	1.19	0.55–2.60	
	III	32	14	0.58	0.23–1.48	

Nodal status	Node 0	71	43	Ref		0.63
	Node 1	45	25	0.81	0.38–1.73	
	Node 2	12	6	0.65	0.19–2.22	
	Node 3	16	7	0.51	0.17–1.52	

HR status	ER−/PR−	71	36	Ref		0.21
	ER+ or PR+	72	44	1.53	0.79–2.97	

HER2/neu status	Negative	125	71	Ref		0.76
	Positive	17	9	0.86	0.31–2.36	

HR: hormone receptor; CPM: contralateral prophylactic mastectomy; OR: odds ratio.

**Table 3:** Adjusted and unadjusted cox regression model for overall survival.

Parameter	Level	Total *N*	*N* died	Univariate model			Multivariable model		
				HR	95% CI	*p* value	HR	95% CI	*p* value
Age at diagnosis	Continuous	144	24	0.99	0.95–1.04	0.72	1.00	0.96–1.05	0.87

Diagnosis year	Continuous	144	24	1.22	1.05–1.43	0.01	1.12	0.95–1.33	0.18

Marital status	Married	103	15	Ref		0.31			
	Other	41	9	1.56	0.68–3.56	

Education	<High school	29	6	Ref		0.40			
	≥ High school	102	15	0.66	0.36–1.70	

Race	Nonwhites	30	8	Ref		0.06	Ref		0.04
	Whites	114	16	0.41	0.18–0.97		0.39	0.16–0.97	

Family history of breast cancer	No	72	11	Ref		0.66	Ref		0.44
	Yes	70	13	1.20	0.54–2.67		0.71	0.29–1.71	

Family history of ovarian cancer	No	123	21	Ref		0.79	Ref		0.54
	Yes	19	3	0.85	0.25–2.87		0.65	0.16–2.57	

Stage	I	42	4	Ref		0.04	Ref		0.10
	II	70	10	1.59	0.50–5.07		1.51	0.45–5.10	
	III	32	10	3.82	1.20–12.19		3.41	0.98–11.90	

Nodal status	Node 0	71	10	Ref		0.58			
	Node 1	45	7	1.16	0.44–3.03	
	Node 2	12	3	2.12	0.58–7.71	
	Node 3	16	4	1.94	0.61–6.19	

CPM	No	63	12	Ref		0.43	Ref		0.58
	Yes	81	12	0.73	0.33–1.62		0.79	0.34–1.82	

HR status	ER−/PR−	71	15	Ref		0.16	Ref		0.14
	ER+ or PR+	72	9	0.56	0.25–1.29		0.50	0.20–1.25	

HER2/neu status	Negative	125	23	Ref		0.11	Ref		0.28
	Positive	17	1	0.27	0.04–2.00		0.33	0.04–2.50	

HR: hormone receptor; CPM: contralateral prophylactic mastectomy; HR: hazard ratio.

## Data Availability

Data are available from the corresponding author upon reasonable request.
